# Current practices in neonatal pain management: a decade after the last Italian survey

**DOI:** 10.1186/s13052-025-01896-x

**Published:** 2025-02-14

**Authors:** Paola Lago, Elisabetta Garetti, Patrizia Savant Levet, Immacolata Arenga, Anna Pirelli, Anna Chiara Frigo, Daniele Merazzi, on behalf Pain Study Group of Italian Society of Neonatology

**Affiliations:** 1 Neonatal Intensive Care Unit, Critical Care Department, Ca’ Foncello Regional Hospital, Treviso, 31100 Italy; 2 Neonatal Intensive Care Unit, Women’s and Children’s Health Department, Azienda Ospedaliera-University of Modena, Modena, Italy; 3 Neonatal Intensive Care Unit, Maria Vittoria Hospital, ASL Città Di Torino, Turin, Italy; 4 Neonatal Intensive Care Unit University P.O. Sant’Anna A.O.U., Città Della Salute e della Scienza Di Torino, Turin, Italy; 5 Pediatric - Neonatologist, Milan, Italy; 6 Department of Cardiac, Thoracic and Vascular Sciences, University of Padua, Padua, Italy; 7 Mother’s and Infant’s Department, Valduce Hospital, Como, Italy

**Keywords:** Pain, Newborn and preterm, Analgesic, Sedative, Opioids, Non-pharmacological and pharmacological interventions, Pain assessment

## Abstract

**Background:**

Neonates admitted to neonatal intensive care units (NICUs), as well as maternity nurseries, typically undergo painful invasive procedures during their hospital stay. We aim to report on current bedside analgesia/sedation and pain assessment practices, 10 years after the previous Italian survey.

**Methods:**

This study employed a cross-sectional electronic survey. A 21-item questionnaire was distributed to directors of birth centers and NICUs to ascertain the policy for pain assessment and management in their respective units. A separate questionnaire was dispatched to neonatologists and nurses registered with the Italian Society of Neonatology. They reported on the analgesic strategies implemented for various painful bedside procedures. Both non-pharmacological and pharmacological analgesia interventions, as well as pain assessment, were analyzed. A regression model was utilized to identify factors that predict pain management practices.

**Results:**

Data on pain management practices were collected from the directors of 153 NICUs and birth centers. Of these, 88.9% reported pain control following guidelines and 47.7% confirmed the presence of a local pain specialist promoting pain management in their unit. A minority, ranging from 16.3% to 41.8%, reported the use of a pain scale, a finding corroborated by the 200 doctors and 239 nurses who responded. At least one non pharmacological intervention (i.e., pacifier, sweet solution, or sensory saturation) was reported in 97.8% of the heel lances performed in the NICU and 96.5% in the maternity nursery, meanwhile for intramuscular injections in 73.8% and 70.3%, respectively. Additionally, it was reported that 22.9% of laryngoscopies were still performed without analgesia. Observations were made over 297 mechanical ventilation and 277 non-invasive ventilation courses, with non-pharmacological analgesia administered in 56.4% and 86.9% and the use of analgesic or sedative drugs in 81.7% and 17.1% of the cases, respectively. Furthermore, routine pain assessment was only undertaken in 68.0% and 64.9% of the cases.

**Conclusions:**

We found a largely common propensity among Italian directors, neonatologists, and nurses to perform analgesic interventions for the most frequently encountered invasive neonatal painful procedures, though the practices are still highly variable. The availability of written guidelines and local pain specialists are confirmed as factors that contribute to the proper management of pain. However, pain assessment is still inadequate and urgently needs to be implemented to allow for tailored pain and stress control and prevention in all infants.

**Supplementary Information:**

The online version contains supplementary material available at 10.1186/s13052-025-01896-x.

## Background

Nowadays, pain and stress management in newborns is deemed one of the most practicable strategies for enhancing neuroprotection, particularly in preterm infants. Numerous individualized care programs aimed at bolstering neurodevelopment, alongside neuroprotection bundles, highlight recommendations to decrease stress and pain from the multitude of invasive procedures infants undergo during their hospital stays [1–3].

Exposure to uncontrolled and repetitive pain in both term and preterm newborns can impact their pain perception later in infancy [4] and impair neurodevelopmental outcomes, including cognition [5], motor function [6], and brain development [7, 8]. Therefore, every professional must implement best practices to mitigate the adverse effects of invasive painful procedures and to continuously monitor for signs of pain. Doing so can help reduce abnormal sensory inputs and stressors, which significantly impact neuronal development and synaptogenesis [9–12].

Since the early 2000s, scientific societies and consensus groups on newborn pain control and prevention have recommended the use of both pharmacological (PhA) and non-pharmacological analgesia (NPA) for all painful and stressful neonatal procedures. Routine pain assessments (PAs) have been suggested to tailor pain interventions appropriately [13, 14]. To understand the implementation of pain management and assessment into clinical practice, researchers have previously conducted surveys [15]. In 2005 and 2013, we investigated this issue at Italian neonatal intensive care units (NICUs). Despite nearly a decade of progress, the use of A/S had improved but was variable, and routine pain assessment was still inadequate [16–18].

Now, ten years later, we are interested in understanding how these practices have changed. To our knowledge, this survey is the first Italian study that prospectively investigates bedside analgesia and sedation (A/S) and PA practices for invasive procedures performed in NICUs and maternity nurseries. It offers a more precise snapshot of these practices. The primary objective of this study was to determine how pain is assessed and treated by caregivers working in neonatology concerning the most frequently painful procedures performed in everyday practice. Moreover, we sought to understand the thoughts of NICU directors regarding pain management and assessment in their units. Lastly, we aimed to identify barriers to implementing A/S interventions and factors for further improving pain management, as they are fundamental aspects of neuroprotection.

## Methods

### Study design and participants

This survey was developed by the Neonatal Pain Study Group of the Italian Society of Neonatology (SIN). In May 2022, an electronic questionnaire was distributed, via a website tool (www.surveymonkey.com), to the directors of birth centers and NICUs across Italy. According to the Annual Birth Event Report (CEDAP 2022), licensed by the National Institute of Health (ISS), Italy has 395 birth centers and 118 NICUs. Non-responders received a reminder every 2 weeks, up to a maximum of three times; if no response was received, we made one phone call to the participant. Our questionnaire included on several topics: how pain and stress are approached in their neonatology ward, either according to written guidelines or personal initiative; the presence of a local pain specialist to facilitate the implementation of A/S protocols; the training of directors on the topic; and the non-pharmacological and pharmacological strategies for controlling pain in various procedures. Finally, we sought to determine which validated pain scale is used to assess pain in each unit.

After 2 months, a distinct questionnaire was mailed to neonatologists and nurses registered with the Italian Society of Neonatology. The survey included general information such as city, professional role, presence of local pain specialists, level of care including the presence of NICU, and work shift. Additionally, they were to report pain control interventions consisting of both non-pharmacological (NPA) and pharmacological analgesic (PhA) interventions, as applicable. They also had to report on PAs conducted at the bedside for skin-breaking procedures such as heel lance, intramuscular injection, and central catheter positioning, as well as laryngoscopy, and invasive and non-invasive ventilation performed during their last work shift. Only one procedure per type in the last work shift was collected for individual operators. We chose these painful procedures because they are most frequently performed in birth centers and NICUs and for which existing pain management recommendations are published. Both non-pharmacological and pharmacological pain management interventions along with pain assessment were analyzed. See Appendix 1 for the directors’ and operators’ questionnaires.

Lastly, we examine the obstacles in implementing pain management following guidelines in each response unit. The database was populated directly by completing the online questionnaires.

Since no demographic, clinical or outcome patient data were collected in this survey, ethical approval was not deemed necessary.

### Statistical analyses

The data were evaluated using descriptive analyses. Both non-pharmacological and pharmacological analgesic interventions were expressed in terms of numbers and percentages for the categorical variables. When analyzing, the categorical variables were compared using either the chi-square or Fischer’s exact test.

The A/S interventions, both pharmacological and non-pharmacological, along with the use of pain assessment for each procedure, were associated with three independent variables: the presence of written guidelines, a local pain specialist in the unit, and the professional role of the operator who registered the procedures. Predictors of A/S usage and PA were identified using a univariate logistic regression model. The results of the regression analyses are presented as odds ratios (ORs) with two-sided 95% confidence intervals (CIs). The statistical analyses were conducted with the SAS 9.4 for Windows. (SAS Institute Inc., Cary, NC, USA).

## Results

### Study population

**Fig. 1:**
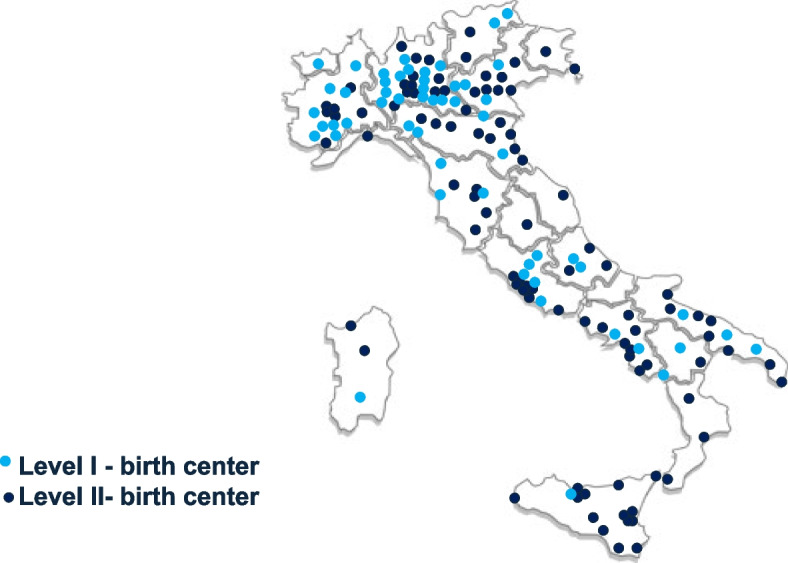
Localization of birth centres responders in the directors Survey’s

Generally, we receive responses from NICUs and birth centers across all regions of Italy. This approach reduces the disparity in response rates between territories, which has been observed in previous surveys (Fig. 1).

### Directors’ survey

Among a total of 395 birth centers’ Directors contacted, responses were received from 153, yielding a response rate of 38.7%. However, this represented 83.0% of the NICU centers (98 out of 118 Birth centers level II with NICUs). Approximately 88.9% of those who responded adhered to written guidelines, either national or local, for various procedures. Besides, 81.7% of directors had undergone specific training on neonatal pain management. The presence of a local pain specialist capable of promoting pain management was declared by 47.7% of the Directors (Table 1).

**Table 1:** Survey of the Directors of the birth centres in Italy (*N* = 153)

Births Centres’ location: *Nord Italy*	78 (51.8%)
* Center Italy *	31 (20.3%)
* Sud Italy*	44 (28.8%)
Neonatal Intensive Care Unit—II Level Birth Centres	98 (64.1%)
Intermedie Neonatal Care—I Level Birth Centres	55 (35.9%)
Pain Control according with writtern guidelines	136 *(*88.9%)
Director’s training on neonatal pain management	125 (81.7%)
Presence of local pain specialist	73 (47.7%)

In general, directors reported strong adherence to written recommendations for pain control and prevention in their wards, albeit with considerable variation depending on the type of painful procedure. The procedures where they reported the lowest likelihood of applying analgesic interventions according with guidelines were intramuscular injections (25.3%) and non-invasive ventilation (28.3%) (Table 2).

**Table 2:** Pain control during Invasive procedure according to the Director’s responses (*N* = 153)

Procedure	SIN Guidelines	Other written Guidelines		No Guidelines	Missing answer*
Heel Prick	108 (70.6%)		38 (24.8%)	7 (4.6%)	0
Venipuncture	105 (68.6%)		36 (23.6%)	12 (7.8%)	0
Arterial Puncture	98 (66.7%)		35 (23.8%)	14 (9.5%)	6
Intramuscular injection	80 (53.3%)		32 (21.3%)	38 (25.3%)	3
ECC positioning	86 (64.7%)		36 (27.1%)	11 (8.3%)	20
Tracheal Intubation	84 (59.6%)		43 (30.5%)	14 (9.9%)	12
Mechanical Ventilation	82 (65.1%)		32 (25.4%)	12 (9.5%)	27
Non-Invasive Ventilation	73 (50.3%)		31 (21.4%)	41 (28.3%)	8
Chest tube insertion	94 (68.8%)		36 (26.3%)	7 (5.1%)	16
Lumbar puncture	99 (67.8%)		40 (27.4%)	7 (4.8%)	7
ROP Screening	68 (49.6%)		46 (33.6%)	23 (16.8%)	16
Therapeutic Hypothermia	70 (64.8%)		27 (25.0%)	11 (10.2%)	45
Post-Operative pain control	58 (64.8%)		36 (33.3%)	14 (13.0%)	45

^*^Percentages are calculated on non-missing values

The use of a pain scale was reported in a maximum of 40.5% of cases for acute pain (DAN, PIPP, NIPS, FLACC), and in 41.8% of cases for prolonged pain (EDIN, COMFORT, N-PASS, FLACC).

### Study population: Doctors’ and nurses’ survey

Furthermore, among those registered with the Italian Society of Neonatology, 200 neonatologists and 239 nurses reported the analgesic strategies they performed concerning invasive procedures conducted at the bedside. Each operator reported only a single procedure done in the last work shift (Table 3).

**Table 3:** Doctor and Nurses—responding to the questionnaire in NICU and maternity birth centers (*n* = 439)

Births Centers in *Nord Italy*	225 51.3%
*Center Italy*	101 (23.0%)
*South Italy*	113 (25.7%)
Doctors	200 (45.6%)
Nurses	239 (54.4%)
Level -II Birth Centres with NICU	382 (87.0%)
Level -I Maternity nursery	57 (13.0%)
Work shift	
morning	196 (50.1%)
afternoon	67 (17.7%)
night	128 (32.7%
Pain control according with written guidelines	337 (76.8%)
Pain control according with individual initiative	102 (23.2%)
Presence of local pain specialist promoting pain management	168 (38.3%)

### Skin-breaking procedures

Heel lances were reported as 364 in the NICU and 228 in the maternity nursery. Manual lancets were still used in 9.7% of procedures performed in NICUs, and 9.0% of those performed in the maternity nursery. At least one NPA intervention (i.e., pacifier, sweet solution, or sensory saturation) was reported in 97.8% of the procedures performed in the NICU and 96.5% in the maternity nursery. Pain assessment was conducted in 61.3% of procedures in the NICU and 46.9% in the nursery.

Intramuscular injections (IMs) were reported as being performed 195 times in NICUs and 165 times in the maternity nursery. NPA practices were reported in 73.8% of NICU cases and 70.3% of nursery cases, respectively.

The analysis of the results demonstrates a statistically significant association between the presence of Level-II NICU birth centers and the utilization of written guidelines for pain management (*p* = 0.037). A similar association is observed between the presence of local pain specialists and the use of those guidelines (*p* < 0.0001). This implies that the application of written protocols for managing pain during skin-breaking procedures is favored in environments that include local pain specialists and NICUs. The presence of a local pain specialist also predicts the use of non-pharmacological interventions, especially for intramuscular injections in NICUs and both heel prick tests and intramuscular injections in maternity nurseries (*p* ≤ 0.05).

In the univariate logistic regression analysis, the use of written guidelines and the presence of a local pain specialist are predictors of pain treatment and assessment performance (Tables 4 and 5).

**Table 4:** Univariate logistic regression models for factors promoting the use of analgesia sedation for heel lance and intramuscolar injection

Predictors of Analgesia/Sedation	Procedure	Univariate	
		***p*-value**	**OR (95% CI)**
Written guidelines for A/S	Heel lance in NICU	0.0026	12.04 (2.38–60.92)
	Heel lance in maternity nursery	0.0014	14.36 (2.79–73.92)
	IM in NICU	< .0001	6.58 (3.21–13.51)
	IM in maternity nursery	0.0056	14.36 (2.79–73.92)
Local Pain specialist	Heel lance in NICU	0.1011	11.03 (0.62–194.59)
	Heel lance in maternity nursery	0.1168	5.419 (0.65–44.79)
	IM in NICU	0.0120	2.59 (1.23–5.47)
	IM in maternity nursery	0.0743	1.907 (0.93–3.87)
Professional Role Doctors/Nurse	Heel lance in NICU	0.7931	1.21 (0.28–5.15)
	Heel lance in maternity nursery	0.3020	2.15 (0.50–9.23)
	IM in NICU	0.0036	0.36 (0.18–0.71)
	IM in maternity nursery	0.9122	1.03 (0.53–2.03)

**Table 5:** Univariate logistic regression models for factors promoting pain assessment

Predictors of Pain Assessment	Procedure	Univariate	
		***p*-value**	**OR (95% CI)**
Written guidelines for A/S	Heel lance in NICU	< .0001	6.58 (2.34–6.77)
	Heel lance in maternity nursery	0.0002	OR 4.43 (2.01–9.74)
	IM in NICU	0.0001	4.53 (2.10–9.78)
	IM in maternity nursery	0.0019	OR 10.34 (2.36–45.24)
Local Pain specialist	Heel lance in NICU	0.0418	1.58 (1.01–2.46)
	Heel lance in maternity nursery	0.0052	2.14 (1.25–3.65)
	IM in NICU	0.0639	1.77 (0.96–3.14)
	IM in maternity nursery	0.2069	1.51 (0.79–2.86)
Professional Role Doctors/Nurse	Heel lance in NICU	0.0924	0.69 (0.45–1.06)
	Heel lance in maternity nursery	0.4873	0.83 (0.49–1.40)
	IM in NICU	0.6269	0.87 (0.49–1.52)
	IM in maternity nursery	0.7425	OR 0.89 (0.47–1.69)

### Other NICU’s painful procedures

In the NICU, 298 instances of central venous catheter positioning were recorded. Non-pharmacological interventions were used in 274 (91.9%) of these cases. This includes the use of a pacifier in 13.1% of cases, a sweet solution in 15.4% of cases, and sensory saturation in 63.4% of cases. Alongside these, pharmacological interventions were employed in 37.7% of cases. Meanwhile, the use of local anesthetic, specifically EMLA, was quite limited, observed in 20.2% of the Central Venous Catheter (ECC) procedures.

The pain scale was used in 43.8% of the procedures to assess pain during ECC positioning, with some instances involving prolonged pain. Tools used included EDIN, FLACC, and N-PASS. The existence of guidelines for ECC positioning favors pain treatment, particularly with NPA interventions (*p* = 0.0034) and pain assessment (*p* < 0.0001). Likewise, the presence of a local pain specialist is correlated with the use of pharmacological interventions for ECC placement (*p* = 0.0029) and with pain assessment (*p* = 0.0053).

**Fig. 2:**
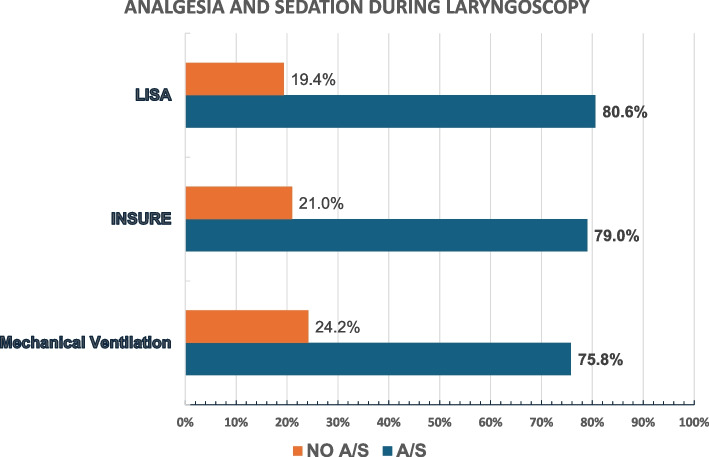
Analgesia and sedation during laringoscopy perphormed for MV, INSURE and LISA

Three hundred and fourteen (314) laryngoscopies were primarily performed to initiate invasive mechanical ventilation (59.6%), but also for INSURE (29.0%) and LISA (11.5%). However, 22.9% of reported laryngoscopies were carried out without A/S. The applied A/S was used at rates of 76.5%, 79.1%, and 80.6% for MV, INSURE, and LISA respectively (Fig. 2). Pain assessment during this procedure was reported in only a minority of cases (34.0%). The existence of written guidelines for tracheal intubation and the presence of a local pain specialist are factors that favor the usage of analgesia and sedation (*p* = 0.0003), as well as pain assessment (*p* = 0.0008).

We observed 297 cases of invasive mechanical ventilation, of which 56.2% received an NPA intervention, and 81.7% were treated with an analgesic or sedative drug. Routine pain assessment was carried out in 68.0% of these cases. Meanwhile, out of 277 non-invasive ventilation courses, 85.9% reported the use of NPA, and 17.1% reported PhA usage, with PAs occurring in 64.9% of instances. A statistically significant association was noted between the availability of written guidelines and the implementation of pain treatment with pharmacological interventions during invasive ventilation sessions (*p* < 0.0001), non-invasive ventilation, and the use of a pain scale for assessment (*p* < 0.0001), as well as the type of pain scale used (*p* = 0.0001). A local pain specialist’s presence correlated significantly with the use of A/S (*p* = 0.0002) and pain-scale assessments in MV (*p* = 0.0024) (Table 6).

**Table 6:** Non-pharmacological and pharmacological interventions during invasive painful procedure

Procedure	NPA	PhA	None	Pain Assessment
Heel Prick (^a^364)	356 (97.8%)	=	8 (2.2%)	223 (61.3%)
Heel Prick (^b^228)	220 (96.5%)	=	8 (3.5%)	107 (46.9%)
Intramuscular injection (^a^195)	144 (73.8%)	=	51 (26.2%)	81 (41.5%)
Intramuscular injection (^b^165)	116 (70.3%)	=	49 (29.7%)	61 (36.9%)
ECC positioning (^a^298)	274 (91.9%)	129 (37.7%)	24 (8.1%)	149 (43.8%)
Laryngoscopy (^a^314)	=	236 (77.1%)	70 (22.9%)	106 (34.0%)
Mechanical Ventilation (^a^297)	167 (56.2%)	237 (81.7%)	NPA130(44.4%) PhA 53 (18.3%)	202 (68.0%)
Non-Invasive Ventilation (^a^277)	238 (85.9%)	47 (17.1%)	NPA 39 (14.1%) PhA 228(82.9%)	180 (64.9%)

*NPA* Non -pharmacological analgesia, *PhA* Pharmacological analgesia

^a^NICU

^b^Nursery

## Discussion

This survey reveals a high level of awareness among NICU and responding birth centers’ directors in Italy regarding practices for mitigating pain and stress in newborns, as well as the use of pain scales for customizing pain interventions. This awareness is particularly evident in NICUs and Level-II birth centers. However, there is a notably low response rate from Level-I birth centers, which could indicate a lack of interest in this matter.

In clinical practice, neonatologists and nurses working at Level-II birth centers and NICUs often implement analgesic interventions, especially for the most common painful procedures, though with some variability. However, pain assessment as a tool to customize pain control interventions is still underutilized.

Skin procedures, specifically heel lances and intramuscular injections, are frequently performed on newborn infants both in maternity nurseries and NICUs [19, 20]. The efficacy and safety of non-pharmacological interventions (such as sweet solutions, sensory saturation, and non-nutritive sucking) have been well-demonstrated, and the level of evidence is moderate to high, making the recommendations strong [21]. In this survey, we noted a very high adherence to best practices in our NICUs, with coverage for heel lance procedures reaching nearly 97.8%, and slightly lower, at 96.5%, in maternity nurseries. However, considering the low response rate from level-I birth centers, our data may not be universally applicable. It is also important to note that our survey identified nearly 10% of heel launches still being performed with manual lancets, even though it is well-established that these tools cause more pain than their automatic counterparts do [22, 23].

While the Italian guidelines for newborn pain management suggest recommendations for intramuscular injection with a low-moderate level of evidence, adherence to pain practices remains low [21]. In fact, for intramuscular injections, the application of non-pharmacological interventions to mitigate pain only occurs 73.8% of the time in NICUs and 70.3% in maternity nurseries. This is a practice that needs to change shortly, given that the procedure is at least as painful as a heel prick.

In previous Italian surveys conducted in 2005 and 2013 by the Pain Study Group, we opted for the standard methodology of interviewing only the director or their representative regarding the clinical practice of analgesia and sedation. Consequently, perhaps the results are less reflective of the reality [16, 18]. Nonetheless, we have documented in those reports that the dissemination of best practices for analgesia and sedation (NPA and PA) at Italian NICUs has improved over time, thereby increasing awareness that A/S interventions are among the neuroprotective interventions to be implemented. The presence of continuously revised national guidelines and training from the Pain Study Group of the Italian Society of Neonatology played an important role in this process. However, more than having a local written guideline, organizational factors such as the presence of a local pain specialist appear to be the more significant factors favoring the implementation of best practices.

To our knowledge, this is the first report that considers the A/S interventions at the bedside during skin-breaking and respiratory invasive procedures in NICUs and maternity nurseries. This provides a more realistic picture of the analgesic and sedative practices employed directly at the patient’s bedside.

Another European survey on A/S practice in NICUs was published in 2015 by Carbajal and his team. However, it focused on the medications used in European NICUs, including Italy, during the first 14 days of admission, rather than on procedures. In this survey, the authors documented that 34% of NICU admissions received analgesic and sedative drugs, primarily those on mechanical ventilation [15].

In a recent Italian survey, the authors distributed a questionnaire to doctors and nurses in five NICUs, inquiring about A/S practices. NPA was used by 64% and 60% of the respondents during heel prick and venipuncture procedures, respectively. Topical analgesia was utilized in 13% of venipunctures, whereas 30% of cases for both heel prick and venipuncture did not involve any analgesia. Regarding pain assessment, 39% reported the absence of pain scales in their departments. These findings underline the recurring issue of inadequate pain management during skin-breaking procedures and the underutilization of pain scales [24].

Most published surveys focus on NICUs, while pain management in maternity units remains poorly documented. A recent survey designed to qualify and quantify clinical practices related to pain assessment and NPA in newborns across Spanish public maternity hospitals found NPA was used in 92.8% of heel pricks and 66.2% of intramuscular injections. However, they documented low usage of pain scales, only employed by 12.5% of the hospitals, with less frequent use of NPA in lower complexity hospitals [19]. As previously noted, we cannot confirm this observation. In our survey, most skin-breaking procedures were recorded in level-II maternity units, where the presence of specialized nurses and neonatologists, with more specific pain training, may make a difference.

ECC placement are typically performed during neonatal intensive care. In our survey, we recorded 298 procedures over a few months, with NPA used in 91.9% of the cases, and analgesic or sedative drugs used in 37.7%. Local anesthesia, mostly with EMLA, was used in 20.2% of the procedures. This data confirms the findings of Bellieni et al. and indicates that awareness about multimodal analgesia in newborns is still lacking.

Laryngoscopy for initiating mechanical ventilation (59.6%) was the primary indication for intubation in our survey, followed by INSURE (29.0%) and LISA (11.5%). It is well-documented that premedication must be used, yet 22.9% of procedures in Italian NICUs are still performed without A/S [25].

In a recent UK survey that used a NICUs’ Director phone interview, the authors discovered that 100% of the NICUs routinely used premedication for babies undergoing tracheal intubation for mechanical ventilation. We need to further enhance the A/S practice during laryngoscopy, particularly for MV, as is accomplished in the UK; this is where we have less concern regarding respiratory depression induced by opioids and sedative drugs used for premedication. Conversely, laryngoscopy for LISA requires heightened attention as the newborn’s respiratory drive should remain efficient. In the UK survey, the authors reported that 57% of the directors claimed to use A/S during LISA. This figure might be improved by selecting drugs with less impact on respiratory drive, such as ketamine [26, 27].

Finally, for both invasive and non-invasive ventilation, we recorded nearly identical usage of A/S to that documented by Carbajal almost 10 years ago: 81.7% for A/S used during MV and 17.1% for non-invasive ventilation. Meanwhile, the use of NPA stood at 56.2% and 85.9%, respectively [15, 28].

In a UK survey on mechanical ventilation, routine use of analgesics and/or sedatives was reported in 54% of cases, while 46% reported that these medications were not used routinely, but only in instances of clear presence of pain and agitation. Just 6% declared that they never use analgesic/sedative medications during mechanical ventilation [26].

NPA interventions need to be more comprehensively implemented, in conjunction with ongoing pharmacological A/S during respiratory assistance. This is a part of multimodal analgesia, as this combined approach should be more efficient than a single pharmacological intervention alone.

Pain assessment is still underutilized in our NICUs and far from being a routine intervention, especially for ongoing and prolonged pain, such as in MV (68.0%) or nINV (64.9%), where pain assessment is fundamental to tailor the analgesic and NPA interventions. We also observed a very low percentage of pain assessment during heel pricks and intramuscular injections performed in the maternity units of Level-I birth centers, at 46.9% and 36.9% respectively. This is slightly better in Level-II units, at 61.3% and 41.5% respectively.

Some pain scales require the monitoring of neonate heart rate, respiratory rate, and O_2_ saturation during the procedure. However, this does not occur routinely, especially in maternity wards. The inclusion of these parameters, for instance in the PIPP scale, hinders the use of these scales. Yet, other behavioral scales for acute pain do not require these parameters, such as DAN and NIPS. Indeed, in a NICU environment, monitoring is routinely performed, making it easier to capture these physiological parameters. Each unit should select the most feasible and affordable pain scale for their setting, whether it be a maternity unit or a NICU, and strive for its consistent use.

In both a Spanish and a French study, the authors reported identical findings: 32% and 43% of the maternity units, respectively, did not conduct PAs primarily because they either found it time-consuming or argued that no pain scale was suitable in this setting [19–23]. In Italy, Law 38/2010, which necessitates PAs for all hospital patients and includes it in the medical chart, should aid this issue, but it appears to still be partly overlooked.

Regarding the barriers hindering the implementation of pain management per guidelines in each respondent’s unit, 32.4% of operators attributed this to cultural factors and a lack of knowledge in pain management. Meanwhile, 31.2% pointed to organizational factors, such as a lack of availability of automatic lancets, sweet solutions, rigid routine activity, and insufficient time dedicated to implementing NPA and PhA. An additional 36.4% identified management issues including the absence of indications, local shared protocols, and difficulties in documentation as barriers. The presence of a local pain specialist could significantly help to overcome these documented barriers, whether they are cultural, organizational, or managerial.

We believe that the strengths of this study include the comprehensive coverage of the entire Italian territory, at least for NICUs, and the methodology that identifies analgesic interventions and monitoring practices directly from caregivers at the patient’s bedside. Understanding what transpires in maternity units, where the majority of newborns are present after birth, is something not undertaken in the past. However, this study is limited by a poor response rate from level-I centers. Another potential issue is that the rate of NPA/PhA use may be overestimated for level-I units, as the numerous non-respondents may have had substandard practices in terms of NPA use and were reluctant to share this information.

Among the factors favoring the use of A/S practices for painful procedures, the presence of a local pain specialist in the unit is more crucial than merely having written guidelines. However, possessing unit written guidelines can be preparatory to implementing analgesia and sedation practices. In a quality improvement process, it is important to identify a local pain specialist who will be responsible for all activities related to pain management.

## Conclusions

We recorded a good awareness of pain management in terms of both pharmacological and NPA interventions among directors of Italian NICUs. However, at the bedside, gaps still exist in implementing the best A/S practices for the most frequently performed painful procedures.

The presence of written guidelines and local pain specialists advocating for the application of sound A/S practices are confirmed factors that should be encouraged. However, pain assessment is still insufficient and urgently requires implementation. This will enable personalized pain and stress control and prevention in all infants.

## Supplementary Information

Supplementary Material 1. Appendix 1 Directors’ and Operators’ questionnaire.

## Data Availability

The datasets generated and analyzed during the present study are available from the corresponding author or DM on reasonable request.

## Abbreviations

A/S: Analgesia/Sedation
PA: Pain Assessment
NPA: Non-Pharmacological Analgesia
PhA: Pharmacological Analgesia
NICU: Neonatal Intensive Care Unit
ECC: Epicutaneous caval catheters
MV: Mechanical ventilation
nNIV: Noninvasive ventilation
SV: Spontaneous ventilation
