# Supplementary material for: Current practices in neonatal pain management: a decade after the last Italian survey
Source: Ital J Pediatr. 2025 Feb 14;51:48. doi: 10.1186/s13052-025-01896-x (PMC11829570; doi:10.1186/s13052-025-01896-x)
Supplement: Supplementary file 1 — Supplementary Material 1. Appendix 1 Directors’ and Operators’ questionnaire. [file 13052_2025_1896_MOESM1_ESM.zip › Appendix 1_Directors Survey Questionaire .docx]

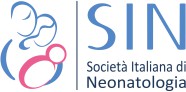

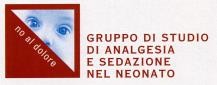
 ***ITALIAN SOCIETY OF NEONATOLOGY***

**Analgesia and sedation study group**

Section 1. GENERAL INFORMATIONS

**CITY**

**Birth Center/Hospital**

**Presence of NICU YES**  **NO** 

Section 2. SPECIFIC INFORMATIONS

1. ***In your unit neonatal, pain control:***
   - ***is carried out according to shared guidelines, recommendations, operating instructions.***
   - ***is left to personal initiative***
2. ***In your professional career you have participated in at least one specific course on neonatal pain***

- ***With faculty members of the GdS - SIN Analgesia and Sedation***
- ***With other organizations -Scientific Society***
- ***Never***

1. ***In your unit there is a local pain specialist -a contact person for pain control?* Yes ⬜** **No ⬜**
2. **In the painful procedures below, if performed in your Unit, what indications for pain prevention and treatment do you implement?**

| ***PAINFUL PROCEDURES*** | ***Nothing*** | ***We follow Guidelines GdS-SIN*** | ***We follow Unit’s Guidelines*** | ***Other Guidelines*** |
| --- | --- | --- | --- | --- |
| ***Heel Prick*** |  |  |  |  |
| ***Venous Puncture*** |  |  |  |  |
| ***Arterial Puncture*** |  |  |  |  |
| ***Central Venous catheter positioning*** |  |  |  |  |
| ***Intramuscular injection*** |  |  |  |  |
| ***Laryngoscopiy*** |  |  |  |  |
| ***Mechanical ventilation with endotracheal tube*** |  |  |  |  |
| ***Non-Invasive Ventilations with nasal cannula/ prongs*** |  |  |  |  |
| ***Lumbar Puncture*** |  |  |  |  |
| ***Pleural drainage*** |  |  |  |  |
| ***Screening for ROP*** |  |  |  |  |
| ***Post Operative pain*** |  |  |  |  |

***Multiple choice possibilities***

| ***Algometric scale for acute procedural pain*** | **DAN** | **PIPP** | **NIPS** | **FLACC** | **Other** |
| --- | --- | --- | --- | --- | --- |
| ***Algometric scale for prolonged or chronic pain*** | **EDIN** | **COMFORT** | **FLACC** | **ALTRO** |  |
