# Supplementary material for: Current practices in neonatal pain management: a decade after the last Italian survey
Source: Ital J Pediatr. 2025 Feb 14;51:48. doi: 10.1186/s13052-025-01896-x (PMC11829570; doi:10.1186/s13052-025-01896-x)
Supplement: Supplementary file 1 — Supplementary Material 1. Appendix 1 Directors’ and Operators’ questionnaire. [file 13052_2025_1896_MOESM1_ESM.zip › Appendix 1_Operators Survey QUESTIONNAIRE.docx]

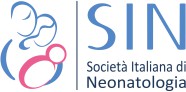

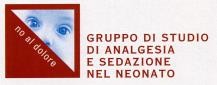


SECTION 1. GENERAL INFORMATIONS

**CITY**

**BIRTH CENTER-HOSPITAL**

**NICU PRESENCE YES**  **NO** 

**PROFESSIONAL ROLE**

- ***Neonatologist-medical doctor***
- ***Nurse-Midwife-Pediatric Nurse-***

SECTION 2. SPECIFIC INFORMATIONS

1. ***In your unit neonatal pain control:***
   - ***is carried out according to shared guidelines, recommendations, operating instructions.***
   - ***is left to personal initiative***
2. **In your unit there is a local pain specialist -a contact person for pain control ?**

**Yes  No **

SECTION 3. DETECTION OF THE PAINFUL PROCEDURE

***INSTRUCTION FOR THE COMPILATION***

This second part will ask for punctual information related to pain management during the following painful procedures

- Heel puncture for screening metabolic tests

- Intramuscular puncture for administration of Vitamin K

- Non-emergency laryngoscopy for tracheal intubation

- Placement of central venous catheter percutaneously

The questionnaire is divided into 2 sections: Maternity Nursery and NICU-Neonatal Pathology. It is required to be completed in relation to the section you belong to.

 For Heel Prick and Intramuscular Puncture, the questionnaire refers to your last shift with date and shift indicated.

 For Intubation and Cannulation it refers to the last procedure performed or observed.


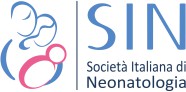

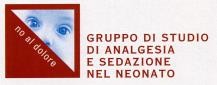


MATERNITY NURSERY

The questions below refer to the last work shift.

Date .........../…….... Work shift: Morning  Afternoon . Night 

| ***HEEL PRICK for METABOLIC SCREENING*** | ***INTERVENTION*** |
| --- | --- |
| ***DEVICE USED*** |  |
| - ***Automatic lancet (Tenderfoot, Gentleheel, Steriheel, Neatnick)*** - ***Other automatic lancet*** - ***Manual lancet or needle*** |      |
| ***ANALGESIC STRATEGY*** |  |
| ***During Breastfeeding*** |  |
| ***Sensorial Saturation (containment + sweet solution ≥ 20% + pacifier + cuddle)*** |  |
| ***Sweet solution (24% Sucrose or 20-33% dextrose)*** |  |
| ***Pacifier and/or containment without sweet solution*** |  |
| ***NO Analgesic measure listed above*** |  |
| ***PAIN ASSESSMENT*** |  |
| ***PAIN ASSESSMENT with algometric scale (DAN, PIPP, NIPS, FLACC, other)*** |  |

| ***INTRAMUSCULAR INJECTION for VITAMIN K administration*** | ***INTERVENTION*** |
| --- | --- |
| ***During breastfeeding, bonding or skin to skin*** |  |
| ***Sensorial Saturation (containment + sweet solution ≥ 20% + pacifier + cuddle)*** |  |
| ***Sweet solution (24% Sucrose or 20-33% dextrose)*** |  |
| ***Pacifier and/or containment without sweet solution*** |  |
| ***NO Analgesic measure listed above*** |  |
| ***PAIN ASSESSMENT*** |  |
| ***PAIN ASSESSMENT with algometric scale (DAN, PIPP, NIPS, FLACC, other)*** |  |


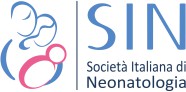

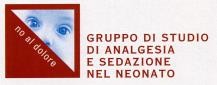
 NICU

The questions below refer to the last work shift.

Date .........../…….... Work shift: Morning  Afternoon  Night 

| ***HEEL PRICK for METABOLIC SCREENING*** | ***INTERVENTION*** |
| --- | --- |
| ***DEVICE USED*** |  |
| - ***Automatic lancet (Tenderfoot, Gentleheel, Steriheel, Neatnick)*** - ***Other automatic lancet*** - ***Manual lancet or needle*** |      |
| ***ANALGESIC STRATEGY*** |  |
| ***During Breastfeeding*** |  |
| ***Sensorial Saturation (containment + sweet solution ≥ 20% + pacifier + cuddle)*** |  |
| ***Sweet solution (24% Sucrose or 20-33% dextrose)*** |  |
| ***Pacifier and/or containment without sweet solution*** |  |
| ***No Analgesic measure listed above*** |  |
| ***PAIN ASSESSMENT*** |  |
| ***PAIN ASSESSMENT with algometric scale (DAN, PIPP, NIPS, FLACC, other)*** |  |

| ***INTRAMUSCULAR INJECTION for VITAMIN K ADMINISTRATION*** | ***INTERVENTION*** |
| --- | --- |
| ***During breastfeeding, bonding or skin to skin*** |  |
| ***Sensorial Saturation (containment + sweet solution ≥ 20% + pacifier + cuddle)*** |  |
| ***Sweet solution (24% Sucrose or 20-33% dextrose)*** |  |
| ***Pacifier and/or containment without sweet solution*** |  |
| ***No Analgesic measure listed above*** |  |
| ***PAIN ASSESSMENT*** |  |
| ***PAIN ASSESSMENT with algometric scale (DAN, PIPP, NIPS, FLACC, other)*** |  |

Survey referring to your last procedure performed or assisted

| ***CENTRAL VENOUS CATHETER INSERCTION*** | **INTERVENTION** |
| --- | --- |
| ***Sweet solution (24% Sucrose or 20-33% dextrose)*** |  |
| ***Sensorial saturation (containment + sweet solution ≥ 20% + pacifier + cuddle)*** |  |
| ***Pacifier and/or containment without sweet solution*** |  |
| ***No Analgesic measure listed above*** |  |
| ***Pharmacologic analgesic intervention (fentanyl or morphine or ketamine)*** |  |
| ***Pharmacologic sedative intervention (benzodiazepine or other)*** |  |
| ***Local Anesthetic (EMLA or other)*** |  |
| ***PAIN ASSESSMENT*** |  |
| ***PAIN ASSESSMENT with algometric scale (DAN, PIPP, NIPS, FLACC, N-PASS,other)*** |  |


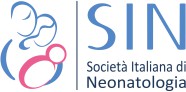

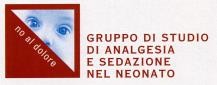
 Survey referring to your last procedure performed or assisted

| ***NON URGENT LARYNGOSCOPY*** |  |
| --- | --- |
| ***INDICATION*** |  |
| ***MECHANICAL VENTILATION***  ***◻ INSURE***  ***◻ LISA***  ***◻*** | |
| ***PATIENT’ CHARACTERISTIC AT THE PROCEDURE*** |  |
| ***Gestational Age <27 ◻ 27-29 ◻ 30-32 ◻ >32 ◻ weeks*** | |
| ***Weight <751 gr ◻ 751-1000 gr ◻ 1001-1500gr ◻ >1500 gr ◻*** | |
| ***Postnatal age (Hours) < 6 h ◻ 6-24 h ◻ 25-48 h ◻ > 48 h ◻*** | |
| ***PHARMACOLOGIC INTERVENTION*** |  |
| ***NO PHARMACOLOGICAL MEASURE*** |  |
| ***ATROPINE*** |  |
| ***OPIOID (Fentanyl, Morphine)*** |  |
| ***CURARE – MUSCLE RELAXANT (Succinylcholine, Mivacurium or other)*** |  |
| ***SEDATIVE (Benzodiazepine)*** |  |
| ***PROPOFOL*** |  |
| ***KETAMINE*** |  |
| ***OTHER pharmacological measure not listed above*** |  |
| ***PAIN ASSESSMENT*** |  |
| ***PAIN ASSESSMENT with algometric scale (DAN, PIPP, NIPS, FLACC, other)*** |  |

| ***INVASIVE MECHANICAL VENTILATION*** |  |
| --- | --- |
| ***PATIENT’ CHARACTERISTIC AT THE PROCEDURE*** |  |
| ***Gestational Age <27 ◻ 27-29 ◻ 30-32 ◻ >32 ◻ weeks*** | |
| ***Weight <751 gr ◻ 751-1000 gr ◻ 1001-1500gr ◻ >1500 gr ◻*** | |
| ***Postnatal age (Hours/Days) < 24 h ◻ 1-7 days ◻ > 7 days◻*** | |
| ***NON-PHARMACOLOGIC INTERVENTION USED*** |  |
| ***Sweet solution (24% Sucrose or 20-33% dextrose)*** |  |
| ***Sensorial Saturation (containment + sweet solution ≥ 20% + pacifier + cuddle)*** |  |
| ***Pacifier and/or containment without sweet solution*** |  |
| ***No Analgesic measure listed above*** |  |
|  |  |
| ***PHARMACOLOGICAL MEASURE: YES***  NO  |  |
| ***FENTANYL***  | Bolus  |
|  | Continous infusion  |
| ***MORPHINE***  | Bolus  |
|  | Continous infusion  |
| ***KETAMINE***  | Bolus  |
|  | Continous infusion  |
| ***MIDAZOLAM***   ***(or another benzodiazepine)*** | Bolus  |
|  | Continous infusion  |
| ***DEXMEDETOMIDINE *** | Bolus  |
|  | Continous infusion  |
| ***OTHER pharmacological measure not listed above; Specify drug and mode of administration*** |  |
| ***PAIN ASSESSMENT*** |  |
| ***PAIN ASSESSMENT with algometric scale (DAN, PIPP, NIPS, FLACC, EDIN, COMFORT, N-PASS, other)*** |  |

| ***NON-INVASIVE ASSISTED RESPIRATORY SUPPORT (WITH NASAL CANNULA OR MASK OR PRONGS)*** |  |
| --- | --- |
| ***PATIENT’ CHARACTERISTIC AT THE PROCEDURE*** |  |
| ***Gestational Age <27 ◻ 27-29 ◻ 30-32 ◻ >32 ◻ weeks*** | |
| ***Weight <751 gr ◻ 751-1000 gr ◻ 1001-1500gr ◻ >1500 gr ◻*** | |
| ***Postnatal age (Hours/Days) < 24 h ◻ 1-7 days ◻ > 7 days◻*** | |
| ***NON-PHARMACOLOGIC INTERVENTION USED*** |  |
| ***Sweet solution (24% Sucrose or 20-33% dextrose)*** |  |
| ***Sensorial Saturation (containment + sweet solution ≥ 20% + pacifier + cuddle)*** |  |
| ***Pacifier and/or containment without sweet solution*** |  |
| ***NO Analgesic measure listed above*** |  |
|  |  |
| ***PHARMACOLOGICAL MEASURE: YES***  NO  |  |
| ***FENTANYL***  | Bolus  |
|  | Continous infusion  |
| ***MORPHINE***  | Bolus  |
|  | Continous infusion  |
| ***KETAMINE***  | Bolus  |
|  | Continous infusion  |
| ***MIDAZOLAM***   ***(or another benzodiazepine)*** | Bolus  |
|  | Continous infusion  |
| ***DEXMEDETOMIDINE *** | Bolus  |
|  | Continous infusion  |
| ***OTHER pharmacological measure not listed above; Specify drug and mode of administration*** |  |
| ***PAIN ASSESSMENT*** |  |
| ***PAIN ASSESSMENT with algometric scale (DAN, PIPP, NIPS, FLACC, EDIN, COMFORT, N-PASS, other)*** |  |

If actions are not put in place in your work to contain the infant's pain during invasive procedures, in your opinion, this is because of reasons

 Cultural (lack of knowledge of the problem)

 Organizational (lack of automatic lancets, lack of or difficulty finding gentle solutions, inadequate space, rigid routine activity, etc.)

 Managerial (lack of precise directions, lack of common recommendations, difficulty in documentation, etc.)

 Other

 (free text space)
